# The Leukemic Phase of ALK-Negative Anaplastic Large Cell Lymphoma Is Associated with CD7 Positivity, Complex Karyotype, *TP53* Deletion, and a Poor Prognosis

**DOI:** 10.3390/cancers13246316

**Published:** 2021-12-16

**Authors:** Lianqun Qiu, L. Jeffrey Medeiros, Guilin Tang, Mahsa Khanlari, Shaoying Li, Sergej Konoplev, Sa A. Wang, C. Cameron Yin, Joseph D. Khoury, Wei Wang, Roberto N. Miranda, Swaminathan Iyer, M. James You, Jie Xu

**Affiliations:** 1 Department of Hematopathology, The University of Texas MD Anderson Cancer Center, Houston, TX 77030, USA; lqiu@mdanderson.org (L.Q.); ljmedeiros@mdanderson.org (L.J.M.); gtang@mdanderson.org (G.T.); Mahsa.Khanlari@STJUDE.ORG (M.K.); sli6@mdanderson.org (S.L.); skonople@mdanderson.org (S.K.); swang5@mdanderson.org (S.A.W.); cyin@mdanderson.org (C.C.Y.); jkhoury@mdanderson.org (J.D.K.); wwang13@mdanderson.org (W.W.); roberto.miranda@mdanderson.org (R.N.M.); mjamesyou@mdanderson.org (M.J.Y.); 2 Department of Lymphoma/Myeloma, The University of Texas MD Anderson Cancer Center, Houston, TX 77030, USA; spiyer@mdanderson.org

**Keywords:** leukemic phase, ALK-negative anaplastic large cell lymphoma

## Abstract

**Simple Summary:**

Anaplastic large cell lymphoma (ALCL) is a systemic peripheral T-cell neoplasm characterized by strong and uniform CD30 expression and, usually, the aberrant loss of one or more T-cell antigens. ALCL is further classified into anaplastic lymphoma kinase (ALK)-positive and ALK-negative types. ALCL rarely involves the peripheral blood. The reported leukemic phase ALCL cases are almost all pediatric patients with ALK-positive ALCL, which are frequently associated with the small cell morphology, t(2;5)(p23;q35), and a poorer prognosis. Leukemic phase ALK-negative ALCL is extremely rare, with approximately ten cases reported in the literature to date, mostly as single case reports. Here we report on nine patients with leukemic ALK-negative ALCL—the largest case series to date—and we compare these cases with 39 non-leukemic cases of ALK-negative ALCL. We show that the patients with leukemic ALK-negative ALCL have a greater frequency of absolute lymphocytosis, thrombocytopenia, bone marrow involvement, CD7 positivity, complex karyotype, *TP53* deletion, and a dismal outcome. These data suggest that leukemic phase ALK-negative ALCL is associated with a number of poor prognostic factors and affected patients may need more aggressive treatment.

**Abstract:**

Patients with anaplastic large cell lymphoma (ALCL) rarely develop a leukemic phase of the disease. The reported leukemic ALCL cases are almost all ALK-positive, which are frequently associated with small cell morphology, t(2;5)(p23;q35), and a poorer prognosis. Rare leukemic ALK-negative ALCL cases have been reported. In the present study, we investigated the clinical and pathologic features and outcomes of nine patients with leukemic ALK-negative ALCL and compared these features with 39 patients without leukemic disease. Compared with the non-leukemic ALK-negative ALCL group, patients with leukemic disease more often had absolute lymphocytosis (50% vs. 0%, *p* = 0.008), thrombocytopenia (60% vs. 11%, *p* = 0.03), bone marrow involvement (50% vs. 14%, *p* = 0.04), and CD7 positivity (71% vs. 19%, *p* = 0.02). Four of five (80%) patients with leukemic ALK-negative ALCL had a complex karyotype, which was significantly higher than that of the patients in the non-leukemic group. A fluorescence in situ hybridization for *TP53* was performed on six leukemic ALK-negative ALCL cases and all (100%) had *TP53* deletion. There were no significant differences in the other clinicopathologic features, treatment, and complete remission rates between patients in the leukemic versus non-leukemic group (all *p* > 0.05). The median follow-up of this cohort was 18 months with a range of 0.3–140 months. Eight of nine (90%) patients with leukemic ALK-negative ALCL died, and their overall survival was significantly shorter than that of the patients with non-leukemic disease (median 15.5 vs. 60 months, *p* = 0.001). In conclusion, we show that the leukemic phase of ALK-negative ALCL is associated with high-risk biologic features and, in particular, a complex karyotype and *TP53* deletion. Compared with the non-leukemic ALK-negative ALCL patients, the patients with a leukemic phase of disease have poorer survival and may require more aggressive treatment.

## 1. Introduction

Anaplastic large cell lymphoma (ALCL) is a systemic peripheral T-cell neoplasm with strong and uniform CD30 expression, which is usually associated with aberrant loss of one or more T-cell antigens. Morphologically, ALCL is characterized by large and pleomorphic cells with horseshoe-shaped nuclei and abundant cytoplasm (so-called “hallmark” cells). The lymphoma cells usually grow in a cohesive pattern, and when nodal architecture is preserved, they can infiltrate sinuses or selectively involve paracortical areas. ALCL is further classified into anaplastic lymphoma kinase (ALK)-positive (+) and ALK-negative types [1]. ALK+ ALCL is characterized by translocations involving *ALK* at 2p23, most commonly t(2;5)(p23;q35)/*NPM1::ALK,* which leads to strong and uniform ALK expression. In the current World Health Organization (WHO) classification, ALK+ ALCL cases are further divided into five variants based on their morphologic features: common, small cell, lymphohistiocytic, Hodgkin-like, and a composite pattern. ALK-negative ALCL is morphologically indistinguishable from the common variant of ALK+ ALCL but lacks the genetic abnormalities involving *ALK* [2]. Patients with ALK-negative ALCL are usually older than those with ALK+ ALCL and have less extra-nodal involvement and a poorer outcome. The long-term overall survival rates of patients with ALK+ and ALK-negative ALCL are 70–90% and < 50%, respectively [3,4,5,6,7,8].

Although B- and T-cell lymphomas can present with or evolve into a leukemic phase of disease, ALCL rarely involves the peripheral blood. The reported leukemic phase ALCL cases are almost all pediatric patients with ALK+ ALCL, which are most often associated with the morphologic features of small cell variant, t(2;5)(p23;q35), and a poorer prognosis [9,10,11,12,13,14]. Leukemic ALK-negative ALCL is extremely rare, with approximately ten cases reported in the literature to date, mostly as single case reports, and the patients had a poor outcome [15,16,17,18,19,20,21]. The clinicopathological features and molecular/genetic abnormalities underlying the leukemic phase of ALK-negative ALCL are largely unknown.

In this study, we identified 48 patients with systemic ALK-negative ALCL whose peripheral blood specimens were examined. Nine patients had a leukemic phase of the disease, either at diagnosis or during the disease course. The clinicopathological features and outcome of these leukemic ALK-negative ALCL patients were examined and compared with their non-leukemic counterparts.

## 2. Materials and methods

### 2.1. Case Selection

One hundred and fifty-six patients with systemic ALK-negative ALCL were identified (1 January 2007–31 December 2020) in the database of The University of Texas MD Anderson Cancer Center. The ALCL cases were diagnosed and subclassified based on the 2016 WHO classification [1]. Among them, the peripheral blood specimens of 48 patients were examined by morphologic evaluation and/or flow cytometric immunophenotyping, 9 of whom were found to have lymphoma cells (leukemic phase disease). The clinical information was collected by reviewing medical records. This study was performed under a protocol (PA16-0897) approved by the Institutional Review Board at the MD Anderson Cancer Center (Houston, TX, USA).

### 2.2. Immunophenotypic Analysis

Immunohistochemistry was performed on formalin-fixed paraffin-embedded (FFPE) tissue sections as described previously [22]. The antibodies used were specific for the following: CD2 (Leica Biosystems, Newcastle, UK); CD7 (Leica Biosystems, Newcastle, UK); EMA (Leica Biosystems, Newcastle, UK); CD3 (Dako, Carpinteria, CA, USA); CD4 (Cell Marque, Rocklin, CA, USA); CD5 (SP4; Labvision/Neomarkers, Fremont, CA, USA); CD8 (Thermo Fisher, Waltham, MA, USA); CD20 (Dako, Carpinteria, CA, USA); CD43 (Dako, Carpinteria, CA, USA); CD45 (Dako, Carpinteria, CA, USA); CD138 (Dako, Carpinteria, CA, USA); ALK (Cell Signaling, Danvers, MA, USA); granzyme B (Thermo Fisher, Waltham, MA, USA); PAX5 (Transduction Labs, San Diego, CA, USA); and p53 (monoclonal, clone DO-7, Dako, Carpinteria, CA, USA).

A flow cytometric analysis was performed on the peripheral blood, bone marrow aspirates, or cell suspensions of the tissue, as described previously [23], using the specific monoclonal antibodies (Becton-Dickinson Biosciences, San Jose, CA, USA) for the following: CD2, CD3, CD4, CD5, CD7, CD8, CD10, CD19, CD20, CD25, CD30, CD45, T-cell receptor (TCR) alpha/beta, and TCR gamma/delta.

### 2.3. Conventional Cytogenetic Analysis

A conventional cytogenetic analysis was performed on the peripheral blood, bone marrow aspirates, or cell suspensions of the tissue, as described previously [24]. The 2020 International System for Human Cytogenetic Nomenclature (ISCN 2020) was used to report the results. Twenty metaphases were analyzed. A complex karyotype was defined as 3 or more abnormalities.

### 2.4. Fluorescence in Situ Hybridization

The fluorescence in situ hybridization (FISH) was performed on the bone marrow, peripheral blood smears, cultured cells, or FFPE tissue sections. The FISH probes used included the *ALK* dual color break-apart probe (Abbott Molecular, Des Plaines, IL, USA), *TP53/CEP17* dual color probe (Abbott Molecular, Des Plaines, IL, USA), and *IRF4/DUSP22* dual color break-apart probe (Cytotest, Rockville, MD, USA). Two hundred interphase nuclei were analyzed [25].

### 2.5. Statistical Analysis

The Graph-Pad Prism 8 was used for statistical analyses. The clinical and pathologic features between leukemic and non-leukemic groups in patients with ALK-negative ALCL were compared using a Fisher’s exact test. The date of the initial diagnosis to the date of death (or last follow-up if patients were alive) was used to calculate the overall survival (OS). The Kaplan–Meier method was used to analyze survival and the log rank test was used to compare survival. A statistical significance was considered if the *p* value was <0.05.

## 3. Results

### 3.1. Clinical Findings

The clinical features of nine patients with leukemic ALK-negative ALCL at time of initial diagnosis are summarized in Table 1 and Table 2. There were seven men and two women with a median age of 61 years (range, 21–74 years). Five of six (83%) patients had B symptoms. Lymphadenopathy was identified in five (56%) patients, and four (44%) patients had extra-nodal involvement. Bone marrow was involved in four of eight (50%) patients assessed (Table 1 and Table 2). Eight patients were fully staged, and all had stage III or IV of the disease. Two of five (40%) patients had leukocytosis and two of four (50%) had absolute lymphocytosis. Four of five (80%) patients had anemia and three of five (60%) had thrombocytopenia. The serum lactate dehydrogenase (LDH) levels were elevated in all three patients tested. Five patients had a known Eastern Cooperative Oncology Group-Performance Status (ECOG-PS) and none of them had a high ECOG-PS. The International Prognostic Index (IPI) score was available for six patients: five had a high IPI score (≥3), and one had a low or intermediate IPI score (<3). The onset of the leukemic phase of the disease was variable. Five (56%) patients were found to have circulating lymphoma cells at initial diagnosis, whereas four (44%) patients developed the leukemic phase at a median interval of 11 months after initial diagnosis. In six patients, the percentage of lymphoma cells in the peripheral blood was available. The median percentage of lymphoma cells was 6.5% (range, 2–75%).

The non-leukemic group included 30 men and 9 women with a median age of 60 years (range, 36–79 years). Twenty-three of thirty-seven (62%) had B symptoms, twenty-five (66%) had lymphadenopathy, and twenty-seven (69%) had extra-nodal disease. Bone marrow involvement was found in 5 of 37 (14%) patients assessed and 28 (72%) patients had stage III or IV of the disease. Thirteen of thirty-six (36%) patients had leukocytosis, and none of these patients had lymphocytosis. Twenty-six of thirty-six (72%) patients had anemia, four of thirty-six (11%) showed thrombocytopenia, and fifteen of twenty-nine (52%) had an elevated serum LDH level.

Comparison of the leukemic versus non-leukemic ALK-negative groups revealed that the former group had an increased frequency of bone marrow involvement (50% vs. 14%, *p* = 0.04), absolute lymphocytosis (50% vs. 0%, *p* = 0.008), and thrombocytopenia (60% vs. 11%, *p* = 0.03). No significant differences were observed in other clinical features between these two groups (all *p* > 0.05). 

### 3.2. Morphologic and Immunophenotypic Findings

**Figure 1:**
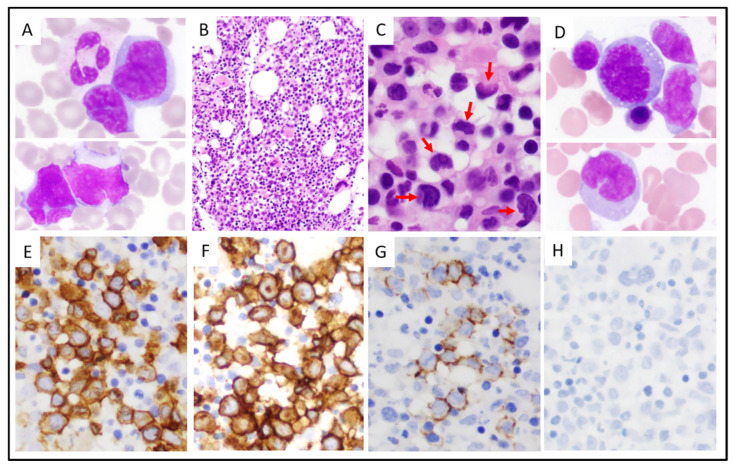
A representative case of leukemic ALK-negative ALCL (case 9). (**A**), The peripheral blood smear showed circulating lymphoma cells with large, pleomorphic nuclei, condensed chromatin, and small to moderate amounts of cytoplasm. (**B**,**C**), The bone marrow core biopsy specimen revealed hypercellular bone marrow involved by lymphoma cells in an interstitial pattern (indicated by arrows). Some of the lymphoma cells had kidney-shaped nuclei (hallmark cells). (**D**), Bone marrow aspirate smears showed lymphoma cells, similar to those in the peripheral blood smear. (**E**–**H**), The lymphoma cells were positive for CD3 (**E**), CD30 (**F**), EMA ((**G**), small subset), and were negative for ALK (**H**). (**A**,**D**), Wright-Giemsa stain, ×1000. (**B**), hematoxylin-eosin stain, ×100. (**C**), hematoxylin-eosin stain, ×400. (**E**–**H**), immunohistochemistry, ×400.

Morphologically, all ALK-negative ALCL cases in tissue biopsy or excision specimens had morphologic features of the so-called “common pattern” regardless of the presence or absence of blood involvement. A leukemic ALK-negative ALCL case is shown in Figure 1. The circulating lymphoma cells were intermediate to large in size, with irregular nuclear contours, partially dense chromatin, and a small to moderate amount of cytoplasm. The bone marrow biopsy specimens showed large lymphoma cells forming into cohesive sheets or in an interstitial pattern. The lymphoma cells with horseshoe-shaped nuclei (“hallmark” cells) were easily identified. Intravascular tumor cells were observed occasionally. The bone marrow aspirate smears showed lymphoma cells with a morphology similar to the lymphoma cells in the blood smear.

**Figure 2:**
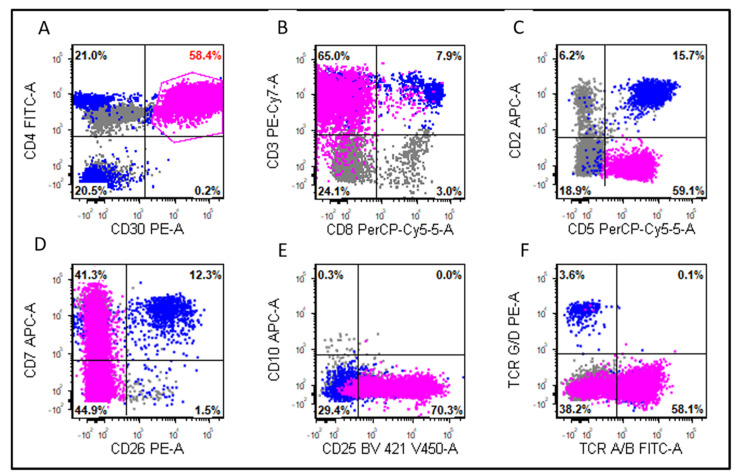
Flow cytometric immunophenotypic analysis of the case shown in Figure 1. The lymphoma cells (pink dots) were positive for CD30 (**A**), CD4 (**A**), CD3 (**B**), CD5 (decreased, **C**), CD7 (partial, **D**), CD25 (**E**), T-cell receptor (TCR) alpha/beta (partial, **F**), and were negative for CD8 (**B**), CD2 (**C**), CD26 (**D**), CD10 (**E**), and TCR gamma/delta (**F**). The blue dots represent background reactive T-cells.

Immunophenotypically, the most frequently expressed T-cell antigens in leukemic cases were CD43 (4/4; 100%), TCR alpha/beta (5/6; 83%), CD3 (7/9; 78%), CD2 (6/8; 75%), CD4 (6/8; 75%), and CD7 (5/7; 71%) (Table 3; Figure 2). Other markers assessed included CD45 (2/2; 100%), EMA (3/5; 60%), CD25 (3/6; 50%), TIA1 (2/4; 50%), granzyme B (1/3; 33%), CD8 (2/8; 25%), and CD5 (1/8; 13%). All the cases tested were negative for TCR gamma/delta (0/4) and CD56 (0/6). The leukemic group showed a significantly higher frequency of CD7 than the non-leukemic group (71% vs. 19%, *p* = 0.02). There were no significant differences in the other markers between these two groups (all *p* > 0.05).

**Figure 3:**
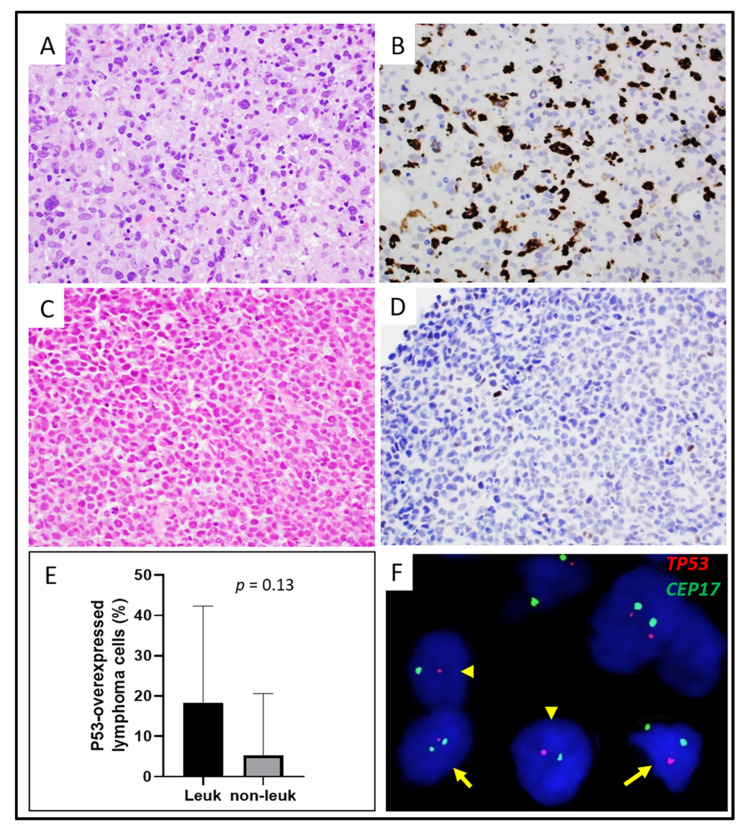
Examination of *TP53* deletion and overexpression. (**A**,**B**), A leukemic ALK-negative ALCL case involving bone marrow (**A**, hematoxylin-eosin stain, ×200) showed p53 protein overexpression (**B**, immunohistochemistry, ×200). (**C**,**D**), A non-leukemic ALK-negative ALCL case involving lymph node (**C**, hematoxylin-eosin stain, ×200) was negative for p53 protein overexpression (**D**, immunohistochemistry, ×200). (**E**), Comparison of p53 overexpression in leukemic versus non-leukemic ALK-negative ALCL cases. (**F**), FISH analysis using *TP53/CEP17* dual color probe (red, *TP53*; green, *CEP17;* ×600) in a leukemic ALK-negative ALCL case showed *TP53* deletion (one red two green, indicated by arrows) or monosomy 17 (one red one green, indicated by arrowheads).

Six leukemic cases had material available for the evaluation of p53 protein overexpression [defined as strong (3+) staining intensity in ≥5% of cells] by immunohistochemistry [26]. The overexpression of p53 was detected in 3 (50%) leukemic cases versus 3 of 18 (17%) non-leukemic cases (*p* = 0.14, Table 3). The mean percentage of lymphoma cells with p53 overexpression was 18% in leukemic cases and 5% in non-leukemic cases, but this difference was not significant (18% vs. 5%, *p* = 0.13; Figure 3). 

### 3.3. Cytogenetic and Molecular Findings

Five leukemic cases had karyotype results available. Four (80%) cases had a complex karyotype, and one case (case #1) showed a normal karyotype (Table 4). Of note, case #1 had a low-level (3%) of lymphoma cells in the specimen assessed by karyotyping, which could have led to a non-representative, “false normal” karyotype. In contrast, only one of seven (14%) non-leukemic cases had a complex karyotype. The difference in the frequency of a complex karyotype in the leukemic group versus non-leukemic groups was significant (*p* = 0.03; Table 3). No recurrent translocations were identified, however, two leukemic cases (#4 and #9) had a translocation involving the chromosome 3q29 locus.

The FISH analysis for *DUSP22* was performed on 5 leukemic and 20 non-leukemic cases (Table 3 and Table 4). One of five (20%) leukemic cases and seven of twenty (35%) non-leukemic cases were positive for *DUSP22* rearrangement (*p* = 1.0, Table 3). The FISH for *TP53* was performed on six leukemic cases and all (100%) showed *TP53* deletion (Table 4; Figure 3). No non-leukemic cases were tested for *TP53* deletion by FISH. Seven non-leukemic cases had karyotype results available and none of them showed deletion of 17p13. A *TP53* mutational analysis was performed on two leukemic cases (Table 4) and one was positive (case #9) for *TP53* mutation. Most of the lymphoma cells in case #9 showed p53 overexpression (Figure 3), which is consistent with a *TP53* mutation (Table 4).

### 3.4. Treatment and Response

All patients with leukemic and non-leukemic ALK-negative ALCL received variable chemotherapy regimens over their disease course, with or without subsequent stem cell transplantation (Table 2). In the leukemic group, seven of nine (78%) patients were treated with cyclophosphamide, doxorubicin, vincristine, and prednisone (CHOP) or modified CHOP. After the initial induction chemotherapy, three of nine (33%) patients with leukemic disease achieved complete remission. Three of nine patients (33%) received brentuximab vedotin. Three of eight (38%) patients received a hematopoietic stem cell transplant: two autologous and one allogeneic.

In the non-leukemic group, 21 of 37 (57%) patients were treated initially with CHOP or modified CHOP. After initial induction chemotherapy, 23 of 36 (64%) patients achieved complete remission. Fifteen of thirty-seven (41%) patients received brentuximab vedotin. Nine of thirty-two (28%) patients underwent hematopoietic stem cell transplant: eight autologous, one allogeneic. There was no significant difference in the treatment or initial complete remission rates between the leukemic and non-leukemic patients (all *p* > 0.05, Table 2).

### 3.5. Outcome

**Figure 4:**
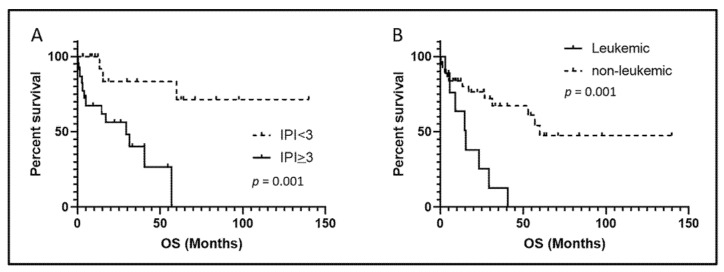
Overall survival (OS) analysis. (**A**), High IPI score (≥3) was associated with shorter OS in all (leukemic and non-leukemic) ALK-negative ALCL patients. (**B**), The patients with leukemic ALK-negative ALCL had significantly shorter OS than the patients with non-leukemic disease. IPI, International prognostic Index.

After a median clinical follow-up of 18 months (range, 0.3–140 months), 21 of 48 (44%) ALK-negative patients (leukemic and non-leukemic) died, including eight of nine (90%) leukemic and thirteen of thirty-nine (33%) non-leukemic patients. The death rate for the leukemic patients was significantly higher than that of the non-leukemic patients (90% vs. 33%, *p* = 0.006). A high (≥3) IPI score was associated with shorter overall survival in all (leukemic and non-leukemic) the patients (*p* = 0.001; Figure 4A). The median overall survival (OS) of the leukemic patients was 15.5 months (range 3.1–40.6 months) versus 60 months (range 0.3–139.9 months) for the non-leukemic patients (*p* = 0.001, Figure 4B).

## 4. Discussion

Peripheral blood involvement by lymphoma (leukemic phase) is highly unusual in ALCL [10], accounting for <5% of all ALCL patients. Most leukemic ALCL cases in the literature were ALK+ and characterized by young patient age (<30 years of age), male predominance, high frequency of B symptoms, extensive extra-nodal disease, small cell morphology, t(2;5)(p23;q35), poor response to chemotherapy, and a poorer outcome [9,10,11,13,14,27]. These case reports also suggest that ALK+ ALCL patients with a complex karyotype may be at a higher risk of developing a leukemic phase of the disease.

Among the patients with leukemic ALK-negative ALCL in this study, more than half had circulating lymphoma cells at the time of the initial diagnosis, whereas the remaining patients developed blood involvement during the disease course. The percentage of the circulating lymphoma cells ranged from 2% to 75% (median 6.5%). The clinical features of the patients with leukemic ALK-negative ALCL in this study were similar to the patients with non-leukemic disease, except that the patients with leukemic disease had a higher frequency of bone marrow involvement, absolute lymphocytosis, and thrombocytopenia. In the literature, ten cases of leukemic ALK-negative ALCL have been reported prior to the current study: eight (80%) patients had leukemic disease at initial diagnosis and two patients developed a leukemic phase five years and twenty years after initial diagnosis, respectively [16,19]. Most of the reported patients also had bone marrow involvement and/or thrombocytopenia [16,17,18,19,20,21].

All the ALK-negative ALCL cases in our study resembled the so-called “common pattern” described for ALK+ ALCL, regardless of blood involvement. Some of the lymphoma cells in the blood or bone marrow aspirate smears had kidney-shaped nuclear contours, similar to the “hallmark” cells, as can be seen in the tissue sections. Most leukemic cases reported in the literature also resembled the common pattern with the exception of one case, in which a small cell morphology was described in a 30-year-old man with leukemic ALK-negative ALCL and had a history of chronic usage of androgenic steroids [15]. Although a small cell morphology is seen in a small subset of ALK+ ALCL cases [28], this variant is very rare and highly challenging to recognize in ALK-negative ALCL.

The immunophenotypic profile of the leukemic ALK-negative ALCL cases was similar to that of the non-leukemic cases except for CD7. About 80% of the non-leukemic ALK-negative ALCL cases in our cohort were negative for CD7 expression, which was consistent with a previous report [29]. In contrast, 71% of leukemic ALK-negative ALCL cases were positive for CD7. The high expression rate of CD7 in leukemic ALK-negative ALCL indicates that CD7 is a potential therapeutic target. CD7 is a transmembrane glycoprotein expressed by T-cells and NK-cells, and is a hallmark molecule for early T-cell differentiation [30]. CD7 is uniformly and strongly positive in T-lymphoblastic leukemia/lymphoma (T-ALL) and remains highly expressed during chemotherapy and at relapse [31,32], and therefore, CD7 is a promising therapeutic target. The chimeric antigen receptor (CAR) T-cells targeting CD7 have showed strong anti-leukemic activity in the T-ALL cell lines and patient-derived xenografts [31]. In a recently reported phase I trial, 20 patients with relapsed/refractory T-ALL treated with anti-CD7 CAR-T-cells achieved a high complete remission rate of 90%, and most of these patients remained in remission with a median follow-up of 6.3 months [33]. A similar approach potentially could be applied to the patients with leukemic ALK-negative ALCL given their poor treatment response and outcome.

Although the mechanisms underlying the leukemic phase of ALK-negative ALCL are unclear, cytogenetic abnormalities may provide a clue. Four of five leukemic ALK-negative ALCL cases in this study had a complex karyotype, which was significantly higher than non-leukemic cases. Most of the previously reported leukemic ALK-negative ALCL cases also had a complex karyotype. Two of four leukemic ALK-negative ALCL cases in this study also had translocations involving chromosome 3q29. Chromosomal translocations have been reported in three leukemic ALK-negative ALCL cases in the literature. One case had a translocation involving chromosome locus 1p36.1 (including *RUNX3)* and this case showed overexpression of RUNX3 [17], one case showed t(2;3)(p21;q21) [20], and one case had an inv(3)(p21q27) [34]. It is intriguing that four leukemic ALK-negative ALCL cases have harbored translocations involving 3q21-29, and it will be of interest to explore the potentially involved gene(s) on this locus.

*TP53*, a tumor-suppressor gene located at chromosome 17p13, is involved in many cell functions including cell cycle arrest, DNA repair, and apoptosis [35]. *TP53* dysfunction is one of the most common abnormalities in various malignancies, including lymphomas. It is known that the p53 protein is expressed frequently in ALCL [36]. This study showed no significant difference in p53 overexpression between the leukemic and non-leukemic ALK-negative ALCL cases. The activity of the p53 protein is often inactivated by *TP53* mutations or oncoproteins-induced suppressive mechanisms in cancer cells. However, *TP53* is not often mutated in ALCL. In an earlier study, <10% of ALCL tumors (ALK+ and ALK-negative) had *TP53* mutations [36]. Recent studies reported *TP53* mutations in 16% of systemic ALCL (11% ALK+, 23% ALK-negative) and 15% of peripheral T cell lymphomas (including ALCL cases); in addition, *TP53* mutation was associated with a poor prognosis [37,38]. These reports suggest that non-mutational mechanisms likely play a role in suppressing the p53 activity in ALCL. In ALK+ ALCL, the NPM-ALK fusion protein can functionally inactivate and/or degrade p53 in a JNK and/or MDM2-dependent manner [39]. Disruption of the p53-MDM2 interaction by nutlin-3a (a small molecule targeting MDM2) activates the p53 pathway resulting in cell-cycle arrest and apoptosis in ALK+ ALCL cells [40]. A recent study revealed a novel repressive mechanism of p53 activity by EBP2 in NPM-ALK-expressing cells [41].

Genomic profiling has shown that the most frequently affected chromosomal regions in ALK-negative ALCL cases are 17p13 and 6q21, the sites of *TP53* and *PRDM1*, respectively. Loss of these regions occur in about 42% and 56% of ALK-negative ALCL cases, respectively [42]. In contrast, the frequency of the losses of 17p13 and 6q21 in ALK+ ALCL is low (each <10%). In the same study, *PRDM1* was shown to be a tumor suppressor gene in ALCL by both in vitro and in vivo experiments. In the current study, a *TP53* FISH analysis was performed on six leukemic ALK-negative ALCL cases and all of them showed *TP53* deletion. This frequency is higher than the reported 17p13/*TP53* deletion rate in ALK-negative ALCL, suggesting a possible association between *TP53* deletion and the leukemic phase of ALK-negative ALCL.

Among the reported leukemic ALK-negative ALCL cases with follow-up data available, six of eight (75%) patients died, most within six months [15,16,17,18,20,34]. In the present study, the overall survival (OS) of the patients with leukemic ALK-negative ALCL was significantly shorter than that of the non-leukemic patients, confirming the poorer outcome of patients with leukemic phase ALK-negative ALCL. The prognostic impact of the IPI score was confirmed in our cohort. A recent study of 235 ALK-negative ALCL patients from the International T-cell Project reported that B symptoms, elevated serum LDH levels, and poor performance status were associated with shorter OS by a multivariate analysis [43]. In our cohort, there were no significant differences in the IPI score, frequency of B symptoms, elevated serum LDH levels, or performance status between the leukemic and non-leukemic groups, suggesting that these prognostic factors do not contribute to the poorer outcome of the patients with leukemic ALK-negative ALCL.

ALK-negative ALCL is a genetically heterogenous entity. About 20–30% of ALK-negative ALCL cases have a *DUSP22*-rearrangement (*DUSP22*-R), which has been reported to be associated with a favorable outcome [5-year overall survival (OS) of 80–90%] [7,44]. In our cohort, *DUSP22*-R was detected in 35% of all the ALK-negative ALCL cases, both leukemic and non-leukemic, consistent with the previously reported rates of *DUSP22-R*. The lack of difference in the frequency of *DUSP22*-R between the leukemic versus non-leukemic ALK-negative ALCL cases suggests that *DUSP22* does not play a role in the different outcomes of these two groups of patients.

The patients with ALK-negative ALCL bearing 17p loss and/or *PRDM1* inactivation had an inferior OS, compared to the patients bearing no 17p loss and *PRDM1* inactivation [42]. Due to the limited size of that cohort, it is unclear whether the inferior OS observed in the ALK-negative ALCL patients could be attributed partially or entirely to *TP53* deletion or *PRDM1* loss. The extremely high frequency of *TP53* deletion in our leukemic ALK-negative ALCL cases suggest that it may contribute to the poor clinical outcome of those patients.

## 5. Conclusions

In summary, we report nine patients with leukemic phase ALK-negative ALCL, the largest case series to date. In this study, patients with leukemic ALK-negative ALCL had a greater frequency of absolute lymphocytosis, thrombocytopenia, bone marrow involvement, CD7 positivity, a complex karyotype, *TP53* deletion, and a poor outcome. Our data suggest that patients with leukemic ALK-negative ALCL may need more aggressive treatment.

## Figures and Tables

**Table 1:** Clinicopathological Features of 9 Patients with Leukemic ALK-negative Anaplastic Large Cell Lymphoma.

Case ID	Age	Gender	WBC (×10^9^/L) ^a^	Hb (g/dL) ^a^	Platelet (×10^9^/L) ^a^	LDH (U/L) ^a^	LN ^a^	BM ^a^	Leukemic Onset (Months) ^b^	Circulating Lymphoma Cell (%)	Overall Survival (Months)	Outcome
1	61	F	n/a	n/a	n/a	n/a	N	n/a	+4	7	5.1	Alive
2	55	M	3.4	10.7	155	627	P	P	0	6	3.1	Dead
3	67	M	6.4	13.4	312	n/a	N	N	+19.4	n/a	23.4	Dead
4	74	M	106.5	11.5	34	7262	P	P	0	75	14.6	Dead
5	62	M	n/a	n/a	n/a	n/a	P	N	+5.8	2	29.5	Dead
6	58	F	n/a	n/a	n/a	n/a	P	N	0	n/a	9.1	Dead
7	21	M	n/a	n/a	n/a	n/a	N	N	+15.2	4	15.5	Dead
8	39	M	9.31	10.4	73	n/a	N	P	0	n/a	5.6	Dead
9	70	M	15.6	15.4	85	5057	P	P	0	37	40.6	Dead

BM, bone marrow involvement; Hb, hemoglobin; LDH, lactate dehydrogenase; LN, lymph node involvement; WBC, white blood cells; P, positive; N, negative; n/a, not available; ^a^ at the time of initial diagnosis; ^b^ time after the initial diagnosis of ALK-negative ALCL, which was set as “0”.

**Table 2:** Clinical Features of Patients with ALK-negative Anaplastic Large Cell Lymphoma (Leukemic vs. non-Leukemic Group).

Clinical Features	Leukemic (*n* = 9)	Non-Leukemic (*n* = 39)	*p* Value
Male: Female	3.5:1 (7/2)	3.3:1 (30/9)	1
Median age, years (range)	61 (21–74)	60 (36–79)	0.8
B symptoms	83% (5/6)	62% (23/37)	0.4
Nodal presentation	56% (5/9)	64% (25/39)	0.71
Extra-nodal involvement	44% (4/9)	69% (27/39)	0.25
Bone marrow involvement	50% (4/8)	14% (5/37)	**0.04**
Stage III or IV	100% (8/8)	72% (28/39)	0.17
Leukocytosis	40% (2/5)	36% (13/36)	1
Absolute lymphocytosis	50% (2/4)	0% (0/35)	**0.008**
Anemia	80% (4/5)	72% (26/36)	1
Thrombocytopenia	60% (3/5)	11% (4/36)	**0.03**
Elevated LDH	100% (3/3)	52% (15/29)	0.24
High ECOG-PS (≥2)	0% (0/5)	6% (2/34)	1.0
High IPI (≥3)	83% (5/6)	51% (18/35)	0.21
Initial treatment			0.13
CHOP or modified CHOP	78% (7/9)	57% (21/37)	0.45
Others	22% (2/9)	43% (16/37)	
Initial CR	33% (3/9)	64% (23/36)	0.14
SCT+	38% (3/8)	28% (9/32)	0.68

Abbreviations: LDH, lactate dehydrogenase; ECOG-PS, Eastern Cooperative Oncology Group-Performance Status; IPI, International Prognostic Index; CHOP, cyclophosphamide, doxorubicin, vincristine, and prednisone; R, rituximab; CR, complete response; SCT, stem cell transplantation.

**Table 3:** Pathologic Features of ALK-negative Anaplastic Large Cell Lymphoma Cases (Leukemic vs. non-Leukemic Group).

Pathologic Features	Leukemic (*n* = 9)	Non-Leukemic (*n* = 39)	*p* Value
**Immunophenotype**			
CD2+	75% (6/8)	74% (20/27)	1
CD3+	78% (7/9)	54% (20/37)	0.27
CD4+	75% (6/8)	75% (24/32)	1
CD5+	13% (1/8)	47% (14/30)	0.11
CD7+	71% (5/7)	19% (5/26)	**0.02**
CD8+	25% (2/8)	14% (4/28)	0.6
CD25+	50% (3/6)	75% (3/4)	1
CD43+	100% (4/4)	79% (11/14)	1
CD45+	100% (2/2)	77% (20/26)	1
CD56+	0% (0/6)	6% (1/16)	1
TCR A/B+	83% (5/6)	60% (3/5)	0.55
TCR G/D+	0% (0/4)	0% (0/4)	1
Granzyme B+	33% (1/3)	60% (9/15)	0.56
TIA1+	50% (2/4)	55% (6/11)	1
EMA+	60% (3/5)	26% (6/23)	0.29
P53 overexpression	50% (3/6)	17% (3/18)	0.14
**Karyotype**			
Complex+	80% (4/5)	14% (1/7)	**0.03**
**FISH**			
*DUSP22*-R+	20% (1/5)	35% (7/20)	1.0

**Table 4:** Cytogenetic and Molecular Findings of 9 Patients with Leukemic ALK-negative Anaplastic Large Cell Lymphoma.

Case ID	Karyotype	*DUSP22-*R (FISH)	*TP53*		
			Deletion (FISH)	Mutation (NGS)	Over-Expression ^a^ (IHC)
1	46,XX[20] ^b^	n/a	P	n/a	P
2	n/a	N	P	n/a	N
3	49~53, XY,−2, del(2)(p13), +3, add(3)(p26), del(3)(p23p24), add(4)(p16), +5, +5, −15, add(17)(q25), −18, −21, +4~8mar[cp20]/	N	P	N	P
4	46, XY, der(3)add(3)(p22)**t(3**;11**)(q29**;q13**)**, t(6;8)(p23;q13), del(13)(q12), add(19)(p13.3)[8]/46, XY[12]	P	P	n/a	N
5	Insufficient for analysis	n/a	n/a	n/a	n/a
6	46, XX[45]/47.XX, −3, add(11)(q23-24), +mar1-2[5]	n/a	n/a	n/a	n/a
7	n/a	N	P	n/a	N
8	n/a	n/a	n/a	n/a	n/a
9	65~68, add(X)(p11.2),−Y, add(1)(p13), del(1)(p13)×2, del(1)(q11.2)×2, der(2;5)(p10;q10), der(3)**t**(1;**3**)(p22;**q29**)×2, −5, del(5)(q13q33), del(6)(q21q25)×2, +7, add(7)(p13)×2, −8, −9, −10, −11, −12, −13, −15, add(16)(q24))×2, der(16)(add(16)(p13.1)add(16)(q12-13), der(17)del(17)(p11.2)del(17)(q11.2), del(17)(p13), i(17)(q10), −19, −20, add(21)(p11.2), +22, +8~9mar[cp12]/46, XY[8]	N	P	P ^c^	P

*DUSP22*-R, *DUSP22*-rearrangement; P, positive; N, negative; n/a, not available; FISH, fluorescence in situ hybridization; IHC, immunohistochemistry; NGS, next generation sequencing; ^a^ defined as strong (3+) staining intensity in >5% of cells; ^b^ only 3% lymphoma cells in the specimen for karyotype; ^c^ variant allele frequency (VAF) = 26.3%.

## Data Availability

The data presented in this study are available within the article.
